# Growth and orientation of copper gallate SURMOFs on cellulosic thin films

**DOI:** 10.1039/d6ra04098e

**Published:** 2026-07-03

**Authors:** Thomas Elschner, Richard Neubert, Nicole Starke, Björn Günther, Felix Plamper, Markus Rüggeberg, Steffen Fischer

**Affiliations:** a Institute of Plant and Wood Chemistry, Dresden University of Technology Pienner Str. 19 01737 Tharandt Germany thomas.elschner@tu-dresden.de +49 351 463-31275; b Institute of Physical Chemistry, TU Bergakademie Freiberg Leipziger Str. 29 09599 Freiberg Germany; c Chair of Forest Utilization, Dresden University of Technology Pienner Str. 19 01737 Tharandt Germany; d Core Facility Environmental Analytics (CFEA), Dresden University of Technology Pienner Str. 19 01737 Tharandt Germany; e Institute of Soil Science and Site Ecology, Dresden University of Technology Pienner Str. 19 01737 Tharandt Germany

## Abstract

Anisotropic surface-mounted metal organic frameworks (SURMOFs) are interesting for electronic, photonic, and sensing applications. Oriented copper gallate films could be obtained by the continuous flow layer-by-layer (LbL) approach from ethanolic solutions. The layer formation of SURMOFs was investigated by quartz crystal microbalance with dissipation monitoring (QCM-D) in real time and crystal orientation was determined by grazing incidence wide angle X-ray scattering (GIWAXS). A deposition of 500 ng cm^−2^ per individual layer was found. For SURMOFs grown on SiO_2_ and cellulose surfaces, the diffraction plane along the pillars of copper octahedra was found to be predominantly oriented parallel to the surface. Cellulosic films possessing catecholic moieties led to complexation of copper ions on randomly oriented polymer chains resulting in SURMOFs without orientation. Thus, the orientation of copper gallate SURMOFs could be controlled by functional cellulosic thin films.

## Abstract 2

Copper gallate SURMOFs grown using a continuous flow layer-by-layer (LbL) approach on SiO_2_ and cellulose surfaces are partially oriented 2D powders, while catecholic moieties result in SURMOFs without orientation.

## Graphical abstract

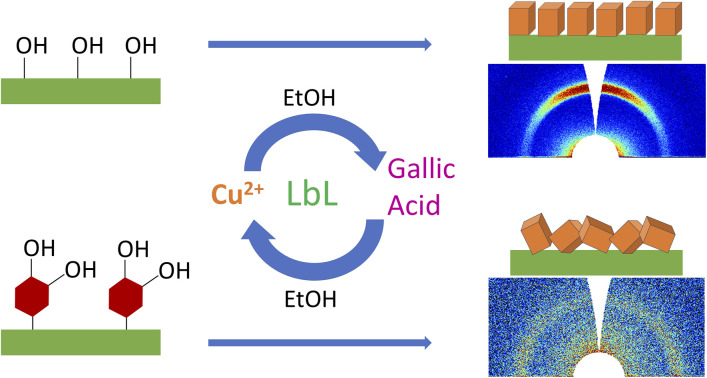

## 1 Introduction

Metal organic frameworks (MOFs) are among the most popular nanomaterials, possessing the highest porosities and adjustable pore sizes. Beyond the storage and separation of gases,^1^ MOFs could be applied in the fields of catalysis^2^ and medicine.^3^ For electronic, photonic, and sensing applications, uniform surface-mounted MOFs (SURMOFs) are crucial and anisotropic effects arising from growth direction become interesting.^4–8^

MOF films can be obtained by casting MOF powders, electrically driven synthesis, and the layer-by-layer (LbL) approach.^9^ The latter approach leads to monolithic, crystalline, uniform, and highly oriented MOF thin films with thicknesses that can be varied in the nanometer to micrometer range.^6^ It is the method of choice for special applications and can be performed in programmed cycles. The LbL method is performed either by chemical vapor^10–13^ or solution deposition.^14–17^ In this manner, the crystal orientation (growth direction) and thus, anisotropy can be controlled by functional surfaces such as metal oxide films, self-assembled monolayers (SAM), and polymer films.

Since copper MOF films of this work were obtained by a continuous flow approach in liquid phase, the following lines focus on the tackling of orientation by LbL technique from Cu(OAc)_2_ solutions. The most popular example is the control of the coordination of the Cu(ii) dimer paddle wheel on the surface by terminated SAM. On the one hand, carboxylic groups can coordinate copper ions, which results in a stand-up orientation. On the other hand, hydroxy groups on the surface coordinate on axial sites of paddle wheels, leading to lying down orientation. Thus, either a [100] or [111] orientation of a HKUST-1 film could be achieved.^15^

Application of different polymer films lead to those orientations, too. HKUST-1 thin films grow along the [111] direction on poly(methyl methacrylate) (PMMA) films, while polyvinyl alcohol (PVA) and nylon 6 films resulted in SURMOFs with [100] orientation.^18^ On polystyrene (PS), no film formation was observed. The authors postulated that hydrogen bonding between copper and oxygen atoms of PMMA and coordination bonding between PVA and nylon 6 films are the reasons.

In context with the aim to use biobased resources and non-toxic ligands, the field of BioMOFs and EL(edible ligands)-MOFs^19^ is developing further, *e.g.* the investigation of gallate-based MOFs.^20–22^ Magnesium- and calcium gallate show antioxidant activity due to the release of gallic acid.^23,24^ Other medical applications were reported for copper gallate (CuGA).^25–28^ In addition to the activity of gallate, the antimicrobial effect of released copper and the loading of the MOF pores with a photosensitizer, was used.

In this work, we intend to explore the potential of CuGA in form of oriented thin films. Up to now, oriented SURMOFs are mostly known for conventional, well-studied Cu- and Zn-based MOFs containing aromatic carboxylate or imidazole-based linkers, *e.g.* HKUST-1, ZIF-8. Unfortunately, the exact unit cell of CuGA is not published and could not be determined within this study, due to the broad, overlapping diffraction lines. Nevertheless, the fundamental structure of CuGA matches a framework with pillars of MO_6_ octahedra connected with linkers to form rhombic channels. The first publication about CuGA by Sharma *et al.*^25^ referred to the structure of MIL-53. The authors applied PXRD (powder X-ray diffraction) and selected area electron diffraction (SAED) to verify the crystal structure and to assign the diffraction planes. However, aluminum terephthalate differs chemically much from CuGA. Considering the family of metal gallates, a similar wine rack structure with rhombic channels is observed.^22^ Different unit cells can be found depending on the metal ion and the crystal phase. In this work, PXRD data of CuGA could not be fitted to any known unit cell and consequently *hkl* planes are not indexed, but there is the evaluable diffraction plane along the pillars of copper octahedra, which possesses an intense reflection.

However, deposition of CuGA on SiO_2_ and cellulose thin films by means of a flow cell was found to be possible. The growth direction can be controlled by functional groups on the surface as revealed by grazing incidence wide angle X-ray scattering (GIWAXS). The MOF films are further investigated by quartz crystal microbalance with dissipation monitoring (QCM-D) and scanning electron microscopy (SEM).

## 2 Experimental section

### 2.1 Materials

Copper(ii) acetate monohydrate (>98% p.a., ACS) was obtained from Carl Roth (Karlsruhe, Germany). Gallic acid (anhydrous) was received from Apollo Scientific (Manchester, UK). Ethanol, absolute (>99.8%) for HPLC-gradient grade was purchased from VWR (Darmstadt, Germany). Trimethylsilyl (TMS) cellulose caffeate (degree of substitution: DS_caffeate_ 0.14, DS_TMS_ 1.72) and TMS cellulose protocatechuate (DS_protocatechuate_ 0.38, DS_TMS_ 1.87) were synthesized according to the literature as published previously.^29,30^ TMS cellulose (DS 2.85) was synthesized as described by Kostag *et al.*^31^

Silicon wafers (P/Bor, 100 mm diameter, 525 µm thickness, [100] orientation) were received from Si-Mat (Kaufering, Germany) and cut into 40 mm square pieces. QCM-D sensors (QSX301 – gold layer and QSX303 – SiO_2_ layer) were obtained from Quantum Design (Pfungstadt, Germany).

Polydimethylsiloxane (PDMS) flow cells were obtained by molding a PMMA master (20 mm × 10 mm × 1 mm) with a degassed mixture of elastomer base and curing agent (Sylgard 184) with a mass ratio of 10 : 1 following the instructions of the supplier. After curing for 3 h at 60 °C, PDMS flow cells were peeled off from the master and perforated with a biopsy punch to enable fluidic inlet and outlet.^32^

### 2.2 Surface pretreatments

Silicon wafers were cleaned with 2-propanol for 10 min in an ultrasonic bath. Afterwards, wafer pieces were dipped into peroxysulfuric acid (piranha solution) for 1 h. Finally, wafers were rinsed with ultrapure water and dried in a nitrogen gas flow.

Wafers with an amorphous SiO_2_ layer (thickness 50 to 100 nm) were obtained by exposition of cleaned wafers to air at 760 °C for 8 h in a muffle furnace.^33^

To obtain cellulosic thin films, silicon wafers and QCM-D sensors were spin coated with silylated polymers dissolved in ethyl acetate (10 mg mL^−1^) at 4000 rpm (acceleration 2500 rpm s^−1^) for 60 s using a POLOS SPIN150i-NPP Single Substrate Spin Processor (Desktop Version) from SPS-Europe B.V. (Putten, Netherlands). TMS groups were cleaved by exposition to hydrochloric acid vapor (from 10 wt% HCl) in Petri dishes for 15 min at room temperature.^30,34,35^

### 2.3 Layer-by-layer coating

**Fig. 1:**
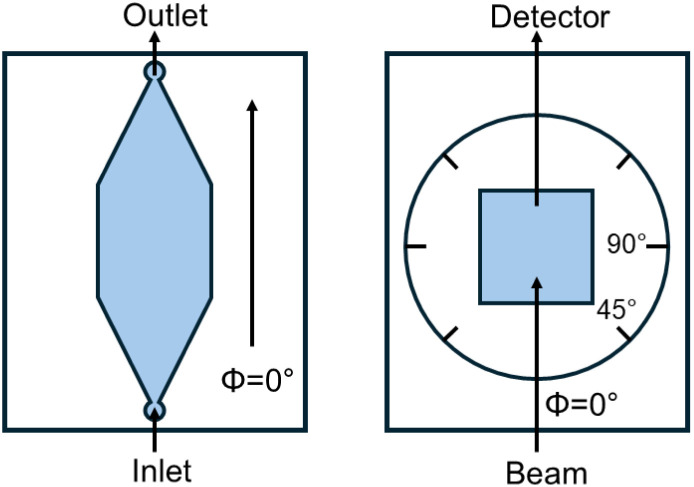
Alignment of the flow cell (left) and the coated wafer on the GISAXS stage (right).

A PDMS flow cell was mounted to a pretreated wafer. The inlet was connected to the outlet of a peristaltic pump, which was sucking from a rotary distribution valve (AMF, Ecublens, Switzerland) to allow automated fluid control. The flow rate was adjusted to 100 µL min^−1^. Coating cycles were carried out with ethanolic copper acetate (0.2 mM) and gallic acid (1 mM) solutions for 10 min, respectively. Between the switch of reagents, rinsing steps with pure ethanol for 20 min were applied. The flow direction of each sample was marked, and the wafers were mounted to the GISAXS stage (with *ϕ*-rotation) along the beam direction at *ϕ* = 0° (Fig. 1).

### 2.4 Measurements

#### 2.4.1 QCM-D

QCM-D measurements were performed with a Q-Sence E4 instrument (Gothenburg, Sweden) at 23.0 °C and a flow rate of 100 µL min^−1^. The four inlet tubes were connected with three Y tubes (0.8 mm) to the outlet of a rotary distribution valve (AMF, Ecublens, Switzerland) to enable automated fluid control. Sensors with a fundamental frequency of *f*_0_ ≈ 5 MHz possessed a sensitivity constant of *C* = 17.7 ng Hz^−1^ cm^−2^. Masses per area were calculated according to Sauerbrey equation, describing a linear relationship between frequency change and adsorbed mass:^36^
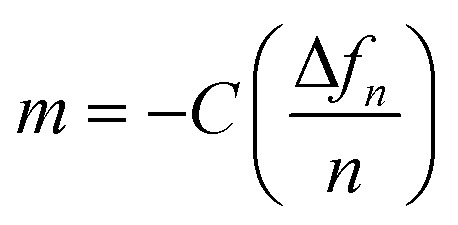


The overtone number (*n*) is considered by the software automatically.

#### 2.4.2 X-ray

Powder XRD of copper gallate was performed on a STOE STADI P diffractometer equipped with a curved Ge (111) crystal monochromator and a Dectris Mythen 1 K detector. For sample fixation, powder was glued between two cellulose acetate films. CuK*α* radiation with a wavelength of *λ* = 0.15418 nm was used and a range of 5° < 2*θ* < 100° with a step size of Δ2*θ* = 0.01° was applied.

Other X-ray experiments were carried out with a SAXSpoint 5.0 laboratory beamline (Anton Paar, Graz, Austria) equipped with a Primux 100 copper microfocus source (CuK*α*, *λ* = 0.15418 nm) and a Dectris EIGER 2R 1M detector without protective foil at 0.4 mbar.

Powder samples were measured with large beam (1 mm) in transmission with WAXS configuration, *i.e.* a sample to detector distance (SDD) of 38.8 mm. Count time was adjusted to 300 s and three frames were averaged. Data were preprocessed with SAXSanalysis software (version 4.50) from Anton Paar applying zero point, beamline parameter fit, and q-transformation tools. LaB_6_ was used as a standard compound for calibration. The background arising from Kapton polyimide tape was subtracted from the samples before reduction to the radial profile.

For GIWAXS experiments, small beam (0.3 mm) was applied and SDD was set to 79.1 mm. The angle of incidence was adjusted to 0.2°. Count time was adjusted to 300 s, recording up to 360 frames. The primary beam was masked for data processing.

Azimuthal integration of the two-dimensional diffraction pattern of the copper gallate (CuGA) films in the region of the diffraction plane at a scattering angle of 2*θ* = 10.2° yielded the azimuthal profiles. Missing data of the azimuthal intensity were completed by fitting with the Voigt function to combine Lorentz- and Gaussian curve using OriginPro 2022 (Fig. S1). The obtained profiles were corrected by subtraction of the background scattering close to the ring. Moreover, azimuthal intensity was weighted with sin *β*, considering the conversion to radian and the range from 0 to π. The degree of orientation (Δ*S*) was calculated according to Cinader & Burghardt,^37^ where *β* is the azimuthal angle and *I*(*β*) is the scattering intensity at angle *β*.
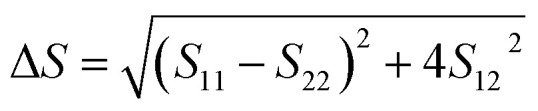

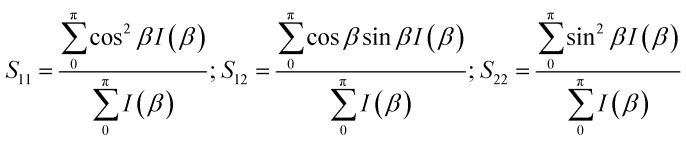


Standard deviations of Δ*S* values were estimated by simulation using the errors of peak width at half-height. Therefore, fit results and noise of azimuthal intensities were considered.

#### 2.4.3 SEM

SEM images were recorded with an ESEM Quanta 650 FEG (FEI) at 10 kV. Samples were fixed on aluminum specimen holders using conductive double sided adhesive carbon tabs.

## 3 Results and discussion

### 3.1 Formation of copper gallate (CuGA)

Originally, CuGA was synthesized by Sharma *et al.*^25^ applying an oil-in-water microemulsion system, which required addition of *N*,*N*-dimethylformamide (DMF). Azhar *et al.*^38^ developed a purely aqueous synthesis procedure, reproduced within this work to yield reference powder material.

CuGA thin films were obtained for the first time by the LbL approach in a polydimethylsiloxane (PDMS) flow chamber or rather in the flow module of a QCM-D device. The setup was adapted from the work of Stavila *et al.*^16^*i.e.* a programmed rotary valve was switched between ethanolic copper acetate solution, ethanolic linker solution, and pure ethanol for the rinsing steps. CuGA SURMOFs could be obtained on silicon wafers and QCM-D sensors.

**Fig. 2:**
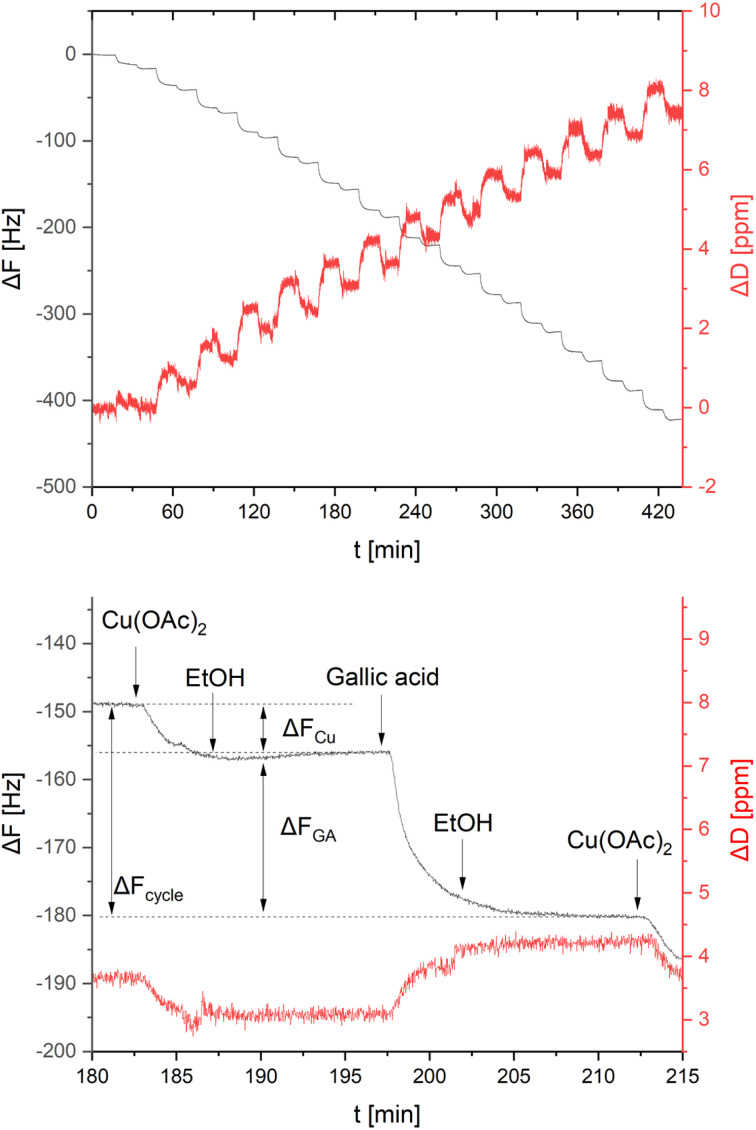
Change in frequency (Δ*F*) and dissipation (Δ*D*) (third overtone) as function of time during SURMOF formation on SiO_2_. Overview on the whole experiment (top) and magnification of one cycle (bottom).

The formation of uniform layers could be monitored by QCM-D in real-time. Fig. 2 (top) shows repetitive even cycles on SiO_2_ coated sensors, indicated by Δ*F* and Δ*D* values over time. A representative cycle starts with the inlet of copper acetate, followed by rinsing with ethanol (Fig. 2, bottom). The deposition of copper ions could be quantified (Δ*F*_Cu_) to compare individual cycles. Subsequently, ethanolic gallic acid is applied, which results in a bigger step marked with Δ*F*_GA_. After rinsing with ethanol, the amount of a single layer can be indicated by Δ*F*_cycle_. The deposition time of copper and gallic acid was set to 10 min in first experiments, but later shortened to 5 min, because there was no difference in deposited masses. The rinsing time of 10 min and reagent concentrations were adapted from literature^16^ describing LbL formation of HKUST-1.

**Fig. 3:**
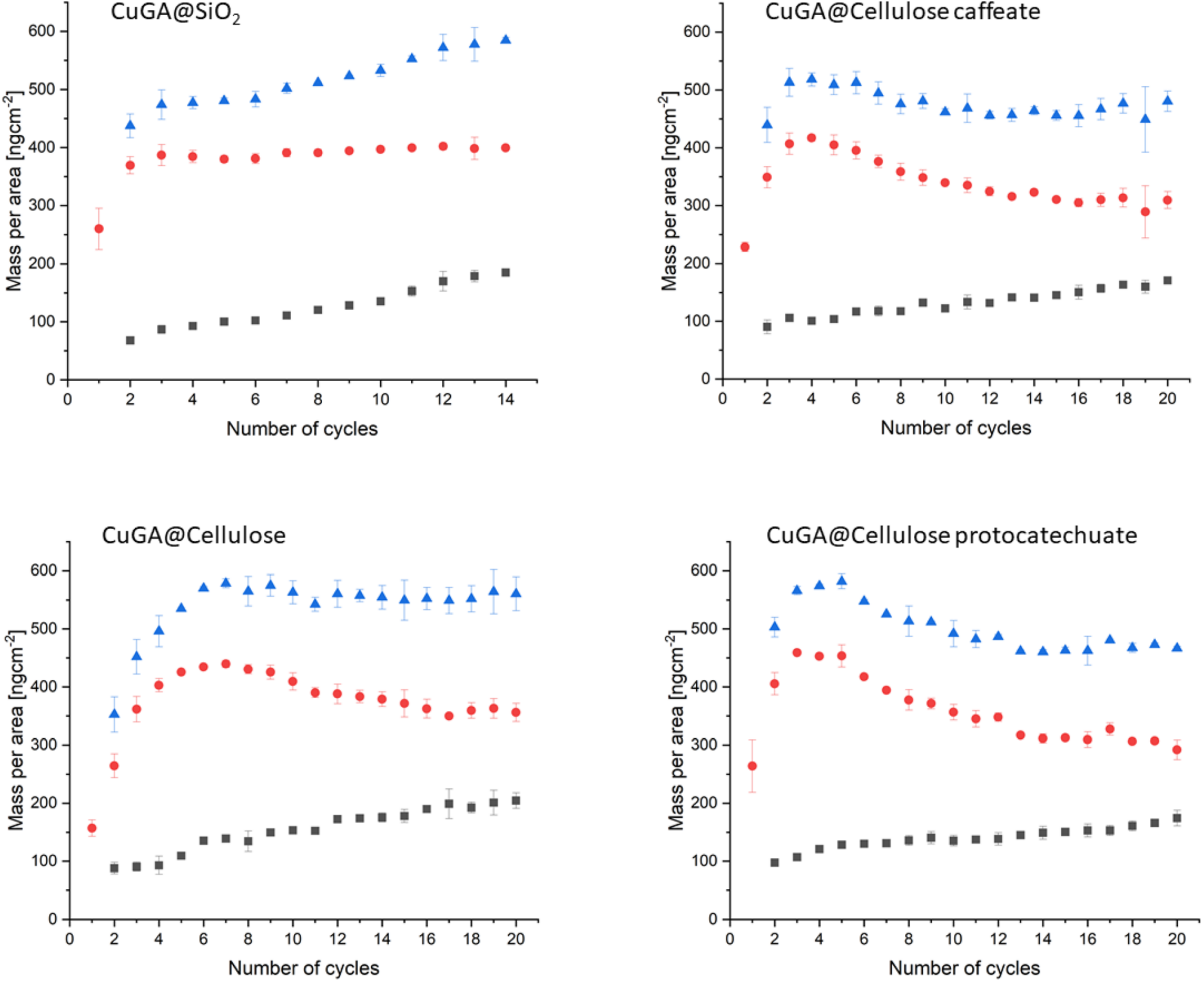
Mass per area of deposited copper acetate (black squares), gallic acid (red circles), and total amount (blue triangles) for individual cycles, calculated from QCM-D data.

Since Δ*F*/Δ*D* is in range of 50 Hz ppm^−1^, *i.e.* layers are rigid, Sauerbrey equation is valid and mass deposition could be quantified. Fig. 3 shows mass per area calculated from Δ*F*_Cu_, Δ*F*_GA_, and Δ*F*_cycle_ for individual cycles. The apparent deposition of gallic acid is significant lower in the first cycle for all support materials. Considering representative complete cycles, values on SiO_2_ surfaces appear periodically with increasing number of cycles with a slight mass increase of copper. It can be assumed that copper ions are not only forming the framework but also deposited in the MOF pores. Alternatively, ethanol, water, or acetic acid could be trapped inside the newly formed framework as proposed for HKUST-1 deposition on QCM-D sensors.^16^

Considering the SURMOF formation on cellulosic films, there is a low adsorption of gallic acid in the first three cycles for pure cellulose films (Fig. 3), but high adsorption of gallic acid for cellulosic films possessing phenolic groups (caffeate, protocatechuate). This can be explained by the pronounced complexation of copper ions by catechol moieties compared to the bare polysaccharide film. Thus, more linker molecules could be bound to surface within one cycle. The high density of anchor points led to faster closure of the surface with CuGA. However, the mass increase from overall MOF growth is similar for all support surfaces and was found to be about 500 ng cm^−2^ per individual layer.

### 3.2 Crystal orientation of copper gallate thin films

The partially amorphous and crystalline structures of CuGA powder depend on the synthesis conditions^38^ and there is no exact unit cell published up to now. Sharma *et al.*^25^ referred to the structure of MIL-53(Al) (aluminum terephthalate)^39^ for characterization by XRD. This MOF is chemically different from CuGA but represents the appropriate model of frameworks with pillars of MO_6_ octahedra connected with linkers to form rhombic channels. When comparing the data of this work with different MIL-53(Al) morphologies from the literature, powder XRD and WAXS measurements suggested the closest similarity with MIL-53lt (low temperature), possessing narrow pore size.^39^ In general, MOFs of the MIL-53 type show large breathing upon hydration. In MIL-53(Al), the hydrogen bonds between water and the oxygen atoms of the framework are responsible for the contraction of the rhombic channels, which lead to a different unit cell and diffraction pattern.

**Fig. 4:**
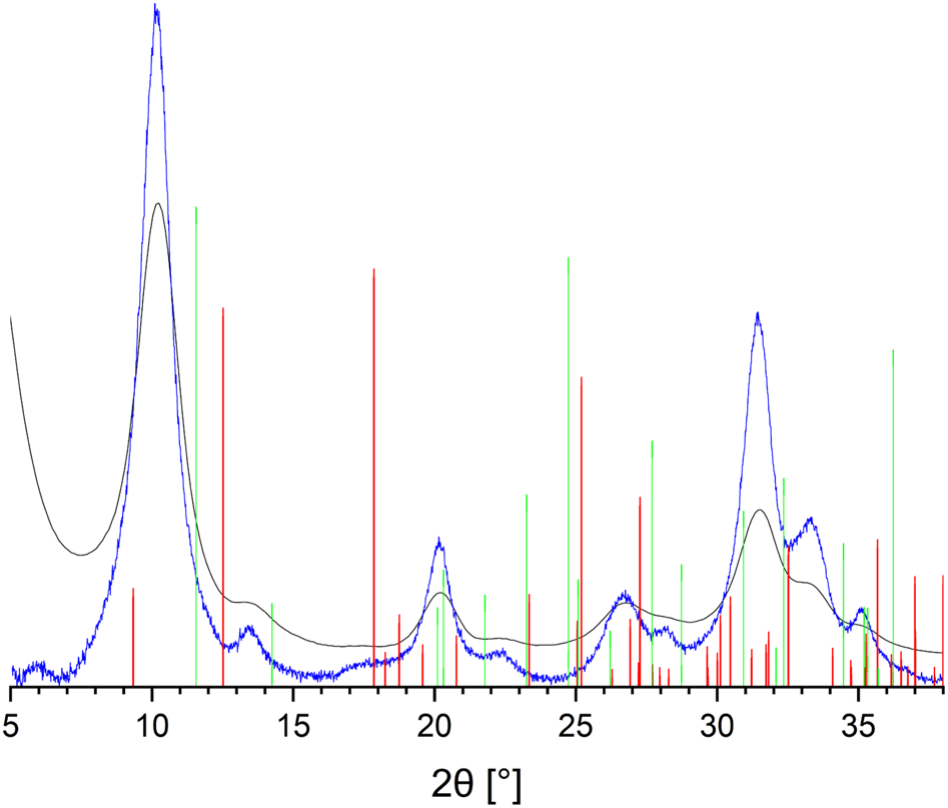
Overlay of the WAXS radial profile of copper gallate (black), powder XRD of copper gallate (blue), and the stick patterns of MIL-53lt^39^(red) and nickel gallate dihydrate^20^ (green).

The reflections to the corresponding planes, radial profiles of the WAXS measurement (black) and the powder XRD (blue) were compared with the stick pattern (red) of MIL-53lt (CCDC 220477)^39^ (Fig. 4). However, no clear assignments were possible.

In further considerations, structures of gallate-based MOFs of the type M(C_7_H_4_O_5_)2H_2_O (M = Mn^2+^, Ni^2+^) with highly defined unit cell^20^ were compared with the data of CuGA. Indeed, there is also the structural motif of infinite chains of corner-sharing distorted MO_6_ octahedra leading to rhombic channels.^21,22^ Due to the similarity of Cu and Ni, XRD data of CuGA were also superimposed with nickel gallate dihydrate, but there was no match (Fig. 4, green). In contrast to nickel, copper octahedra are predestined for the Jahn–Teller effect^40^ and thus, there might be a distortion along the *c*-axis, which lead to ambiguous interpretation of miscellaneous reflections.

Consequently, the unit cell of CuGA remains unknown, and *hkl* planes could not be indexed. For example, it is not clear if the diffraction plane at 2*θ* = 10.2° arises from the (200) plane of the MIL-53lt structure or from the (100) plane of the nickel gallate dihydrate crystal type. However, the intense reflection is related to a plane that is embedding parallel pillars of copper octahedra in any case. Due to the unclear assignment of the other planes, the direction of the pillars relative to the angle *ϕ* could not be determined from GIWAXS experiments.

**Fig. 5:**
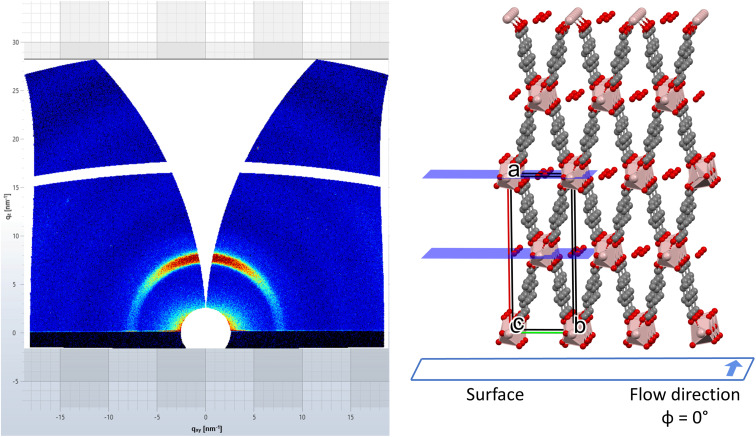
Left: GIWAXS pattern of a copper gallate (CuGA) SURMOF on a pure cellulose film (*ϕ* = 0°). The dominant ring with the maximum indicates anisotropy by the diffraction plane at *q* = 7.5 nm^−1^ (2*θ* = 10.2°). Right: MIL-53lt crystal structure with pillars of octahedra aligned parallel to the flow direction (*c*-axis) connected with linkers, which represents a family of MOFs with rhombic channels. Prominent planes (blue) are oriented parallel to the pillars and the surface. *a*-Axis and *b*-axis of the unit cell are marked in red and green, respectively. Structure was drawn from data of MIL-53lt (CCDC 220477, aluminum terephthalate with narrow pores)^39^ with Mercury software.

Nevertheless, the diffraction plane at 2*θ* = 10.2° of CuGA films (45 cycles) on SiO_2_ and pure cellulose supports is predominantly oriented parallel to the surface. The azimuthal intensity distribution in Fig. 5 (left) clearly indicates this anisotropy by a maximum of the ring assigned to the plane along the pillars of copper octahedra. Moreover, this reflection is almost independent of the *ϕ*-rotation of the sample (Fig. S2). Table 1 shows the degree of orientation (Δ*S*) for *ϕ* from 0° to 90° to the flow direction. It is clearly visible that Δ*S* is about 0.6 for the SURMOF on SiO_2_ and 0.65 on cellulose for any rotation angle.

**Table 1:** Degree of orientation (Δ*S*, according to Cinader & Burghardt^37^) of anisotropic SURMOFs, depending on the rotation angle (*ϕ*) of the sample around the surface normal

Sample	*ϕ* [°]	Δ*S*
SiO_2_	0	0.62 ± 0.01
SiO_2_	30	0.60 ± 0.02
SiO_2_	45	0.61 ± 0.02
SiO_2_	90	0.59 ± 0.02
Cellulose	0	0.65 ± 0.01
Cellulose	45	0.64 ± 0.01
Cellulose	90	0.64 ± 0.01
Cellulose caffeate	0	0.22 ± 0.04
Cellulose protocatechuate	0	0.19 ± 0.03

**Fig. 6:**
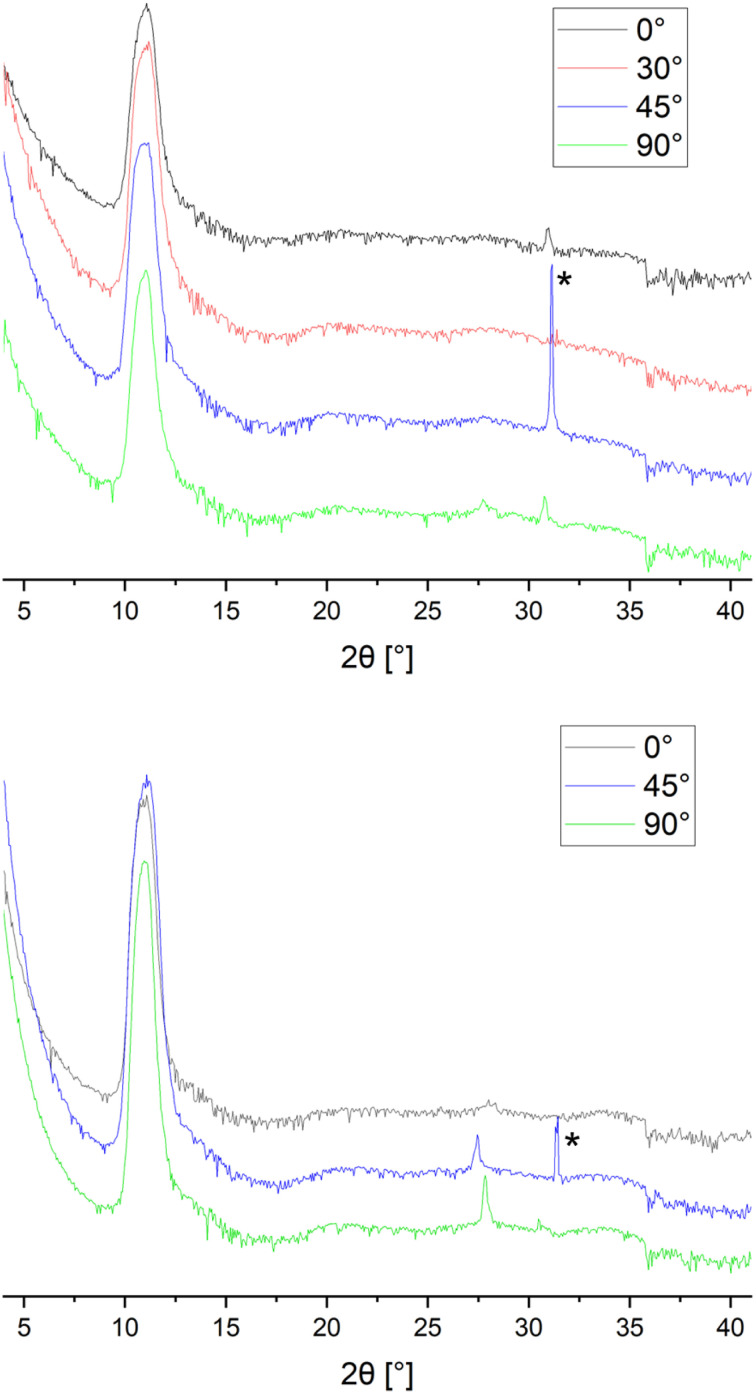
Radial profiles obtained from GIWAXS experiments of copper gallate SURMOFs grown on SiO_2_ (top) and pure cellulose films (bottom), depending on the rotation angle (*ϕ*) of the sample around the surface normal.

Interestingly, there is a significantly higher intensity of the reflection at 31° at *ϕ* = 45° for SURMOFs on SiO_2_ and cellulose, visible in the radial profiles (marked with an asterisk in Fig. 6). Thus, the measurement shows a partially oriented 2D powder.^13^ It could be speculated that the fluidic forces along *ϕ* = 0° lead to an alignment of rhombic channels due to a lower resistance of the flow in the layer (Fig. 5, right). However, this hypothesis is not supported by GIWAXS data.

To conclude, two different effects could be responsible for anisotropy of the material. At first, surface coordination is most likely leading to a parallel orientation of the dominant plane to the surface. It is plausible that hydrogen bonding between copper and oxygen atoms of the polymer film may lead to a stand-up orientation of Cu(ii) dimer paddle wheels, as previously observed for PVA and nylon 6.^18^ However, the occurrence of this mechanism is speculated. The second effect might be related to fluidic forces in the flow chamber. There is an orientation with the direction of *ϕ* visible in the GIWAXS data, but the reflections could not be assigned to any diffraction plane. Thus, the exact orientation and the reasons for this anisotropy are still unknown.

Azimuthal intensity distributions and radial profiles of SURMOFs grown on cellulosic films possessing catechol groups (cellulose caffeate and -protocatechuate) indicate no orientation at all. It is clearly visible in the GIWAXS patterns (Fig. S3). This can be explained by a pronounced complexation of copper ions on catechol groups. Since cellulose chains with phenolic groups are randomly oriented on the polymer film, there is a force towards isotropic formation of SURMOFs.

**Fig. 7:**
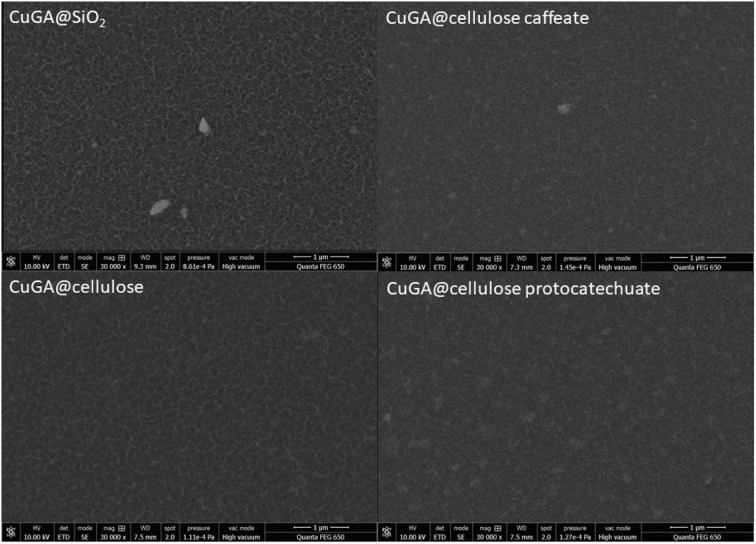
SEM images of SURMOFs at 30k magnification.

The difference between partially and randomly oriented samples observed in GIWAXS experiments is in line with SEM measurements (Fig. 7). Anisotropic SURMOFs on SiO_2_ and cellulose show domains with a size of about 100 nm separated by sharp wrinkles. MOF films grown on cellulose, decorated with catechol groups, appear to be more amorphous with blurred structures.

## 4 Conclusions

SURMOFs of biobased CuGA were deposited by the continuous flow LbL method with a programmed rotary valve applying ethanolic solutions of copper acetate and gallic acid. The layer formation on SiO_2_ and cellulosic thin films was monitored online by QCM-D. As calculated from Sauerbrey equation, the deposited amount of 500 ng cm^−2^ per individual layer was found on all support materials. Moreover, penetration of copper ions into the pores of the framework was detected.

Crystal orientation was revealed for CuGA layers grown on SiO_2_ and bare cellulose films applying GIWAXS. *ϕ*-Rotation of the sample showed that the diffraction plane at 2*θ* = 10.2° was predominantly oriented parallel to the surface. The degree of orientation (Δ*S*) according to Cinader & Burghardt was in the range of 0.6 to 0.65. The occurrence of another reflection at a rotation angle of *ϕ* = 45° showed the presence of a partially oriented 2D powder. It is plausible that pillars of copper octahedra along the rhombic channels were aligned parallel to the flow direction. However, catecholic moieties of cellulose caffeate and -protocatechuate strongly interact with copper ions on randomly oriented polymer chains, which led to SURMOFs without orientation.

Thus, the orientation of CuGA SURMOFs can be controlled by functional cellulosic thin films, which is very interesting for electronic, photonic, and sensing applications. CuGA is composed of the biobased linker gallic acid and a promising alternative to extensively studied MOFs with aromatic carboxylate and imidazole-based linkers, such as HKUST-1 or ZIF-8. To the best of our knowledge, the formation of SURMOFs of copper gallate and their orientation was not described before.

## Author contributions

T. Elschner: conceptualization, funding acquisition, project administration, investigation, data curation, formal analysis, writing – original draft, visualization; R. Neubert: conceptualization, investigation, methodology, resources, data curation, formal analysis, writing – review & editing; N. Starke: investigation, methodology, formal analysis, writing – review & editing; B. Günther: investigation, resources, formal analysis, writing – review & editing; F. Plamper: conceptualization, supervision, resources, writing – review & editing; M. Rüggeberg: supervision, methodology, formal analysis, resources, writing – review & editing; S. Fischer: supervision, resources, writing – review & editing.

## Conflicts of interest

There are no conflicts to declare.

## Supplementary Material

RA-016-D6RA04098E-s001

## Data Availability

The data supporting this article have been included as part of the supplementary information (SI). Supplementary information is available. See DOI: https://doi.org/10.1039/d6ra04098e.

## References

[cit1] Czaja A. U., Trukhan N., Müller U. (2009). Chem. Soc. Rev..

[cit2] Lee J., Farha O. K., Roberts J., Scheidt K. A., Nguyen S. T., Hupp J. T. (2009). Chem. Soc. Rev..

[cit3] McKinlay A. C., Morris R. E., Horcajada P., Férey G., Gref R., Couvreur P., Serre C. (2010). Angew. Chem., Int. Ed..

[cit4] Zacher D., Shekhah O., Wöll C., Fischer R. A. (2009). Chem. Soc. Rev..

[cit5] Stavila V., Talin A. A., Allendorf M. D. (2014). Chem. Soc. Rev..

[cit6] Wang Z., Wöll C. (2019). Adv. Mater. Technol..

[cit7] Rahmati Z., Khajavian R., Mirzaei M. (2021). Inorg. Chem. Front..

[cit8] Tian L., Liu J., Wöll C. (2025). Surf. Sci. Rep..

[cit9] Crivello C., Sevim S., Graniel O., Franco C., Pané S., Puigmartí-Luis J., Muñoz-Rojas D. (2021). Mater. Horiz..

[cit10] Stassen I., Styles M., Grenci G., Gorp H., Vanderlinden W., Feyter S., Falcaro P., Vos D. D., Vereecken P., Ameloot R. (2016). Nat. Mater..

[cit11] Cruz A. J., Arnauts G., Obst M., Kravchenko D. E., Vereecken P. M., De Feyter S., Stassen I., Hauffman T., Ameloot R. (2021). Dalton Trans..

[cit12] Rodríguez-Hermida S., Kravchenko D. E., Wauteraerts N., Ameloot R. (2022). Inorg. Chem..

[cit13] Fischer J. C., Li C., Hamer S., Heinke L., Herges R., Richards B. S., Howard I. A. (2023). Adv. Mater. Interfaces.

[cit14] Shekhah O., Wang H., Kowarik S., Schreiber F., Paulus M., Tolan M., Sternemann C., Evers F., Zacher D., Fischer R. A., Wöll C. (2007). J. Am. Chem. Soc..

[cit15] Shekhah O., Wang H., Zacher D., Fischer R. A., Wöll C. (2009). Angew. Chem., Int. Ed..

[cit16] Stavila V., Volponi J., Katzenmeyer A. M., Dixon M. C., Allendorf M. D. (2012). Chem. Sci..

[cit17] Steinbach D., Neubert R., Gersdorf S., Schimpf C., Erb D., Rafaja D., Plamper F. A., Mertens F. (2023). CrystEngComm.

[cit18] Ohara H., Yamamoto S., Kuzuhara D., Koganezawa T., Oikawa H., Mitsuishi M. (2020). ACS Appl. Mater. Interfaces.

[cit19] Lv D., Nong W., Guan Y. (2022). Coord. Chem. Rev..

[cit20] Feller R. K., Cheetham A. K. (2006). Solid State Sci..

[cit21] Bao Z., Wang J., Zhang Z., Xing H., Yang Q., Yang Y., Wu H., Krishna R., Zhou W., Chen B., Ren Q. (2018). Angew. Chem., Int. Ed..

[cit22] Ismail M., Bustam M. A., Yeong Y. F. (2020). Crystals.

[cit23] Cooper L., Hidalgo T., Gorman M., Lozano-Fernandez T., Simon-Vazquez R., Olivier C., Guillou N., Serre C., Martineau C., Taulelle F., Damasceno-Borges D., Maurin G., Gonzalez-Fernández A., Horcajada P., Devic T. (2015). Commun. Chem..

[cit24] Hidalgo T., Cooper L., Gorman M., Lozano-Fernández T., Simon-Vazquez R., Mouchaham G., Marrot J., Guillou N., Serre C., Fertey P., Gonzalez-Fernández A., Devic T., Horcajada P. (2017). J. Mater. Chem. B.

[cit25] Sharma S., Mittal D., Verma A. K., Roy I. (2019). ACS Appl. Bio Mater..

[cit26] Elmehrath S., Ahsan K., Munawar N., Alzamly A., Nguyen H. L., Greish Y. (2024). RSC Adv..

[cit27] Zong Q., Peng X., Wu H., Ding Y., Ye X., Gao X., Sun W., Zhai Y. (2024). Int. J. Biol. Macromol..

[cit28] Sharma M., Kumar P. (2025). Inorg. Chem. Commun..

[cit29] Elschner T., Schönrich J., Fischer S. (2025). Polym. Sci. Technol..

[cit30] Elschner T., Schönrich J., Bračič M., Maver T., Maver U., Fischer S. (2025). Sci. Rep..

[cit31] Kostag M., Köhler S., Liebert T., Heinze T. (2010). Macromol. Symp..

[cit32] Obst F., Simon D., Mehner P. J., Neubauer J. W., Beck A., Stroyuk O., Richter A., Voit B., Appelhans D. (2019). React. Chem. Eng..

[cit33] Lafatzis D., Mergia K. (2013). J. Appl. Phys..

[cit34] Mohan T., Kargl R., Doliska A., Vesel A., Köstler S., Ribitsch V., Stana-Kleinschek K. (2011). J. Colloid Interface Sci..

[cit35] Elschner T., Adam J., Lesny H., Joseph Y., Fischer S. (2022). Biomacromolecules.

[cit36] Sauerbrey G. (1959). Z. Phys..

[cit37] Cinader Jr. D. K., Burghardt W. R. (1999). J. Polym. Sci., Part B: Polym. Phys..

[cit38] Azhar B., Angkawijaya A. E., Santoso S. P., Gunarto C., Ayucitra A., Go A. W., Tran-Nguyen P. L., Ismadji S., Ju Y.-H. (2020). Sci. Rep..

[cit39] Loiseau T., Serre C., Huguenard C., Fink G., Taulelle F., Henry M., Bataille T., Férey G. (2004). Chem.–Eur. J..

[cit40] Hu K.-L., Kurmoo M., Wang Z., Gao S. (2009). Chem.–Eur. J..

