# Supplementary material for: Growth and orientation of copper gallate SURMOFs on cellulosic thin films
Source: RSC Adv. 2026 Jul 3;16(34):31877–84. doi: 10.1039/d6ra04098e (PMC13331680; doi:10.1039/d6ra04098e)
Supplement: RA-016-D6RA04098E-s001 [file RA-016-D6RA04098E-s001.pdf]

Supplementary Information (SI) for RSC Advances.
This journal is © The Royal Society of Chemistry 2026

Supplementary Information

Growth and orientation of copper gallate SURMOFs on cellulosic thin films

Thomas Elschner, Richard Neubert, Nicole Starke, Björn Günther, Felix Plamper, Markus Rüggeberg, Steffen Fischer*

Figure S1 (a-i): Voigt fits of the azimuthal intensities of the ring at 2θ = 10.2° to complete missing data.

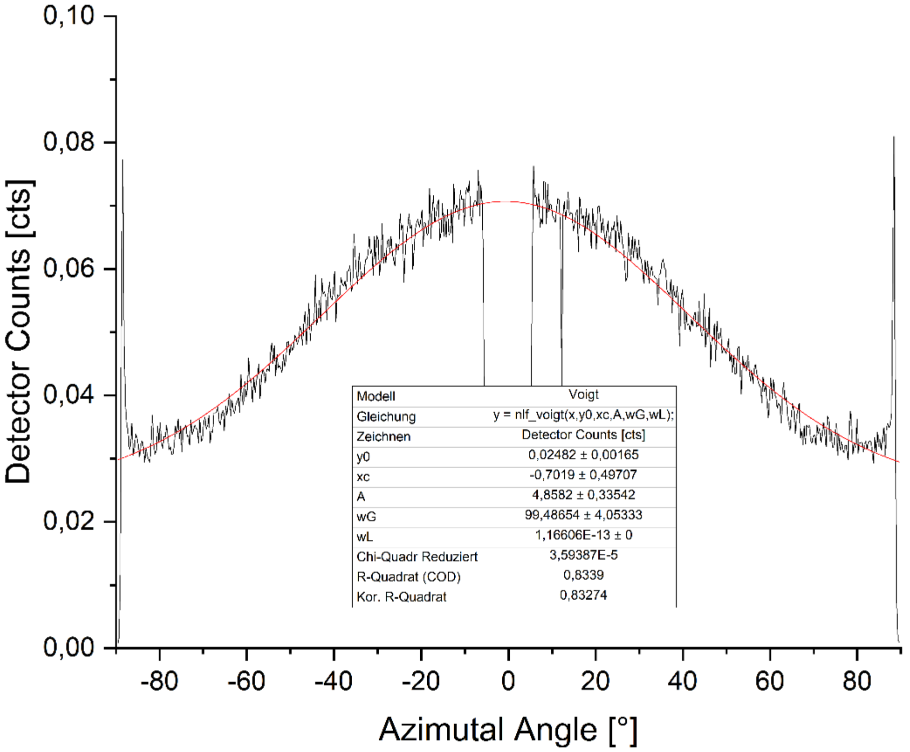

Figure S1a: CuGA@SiO_2, ϕ = 0°

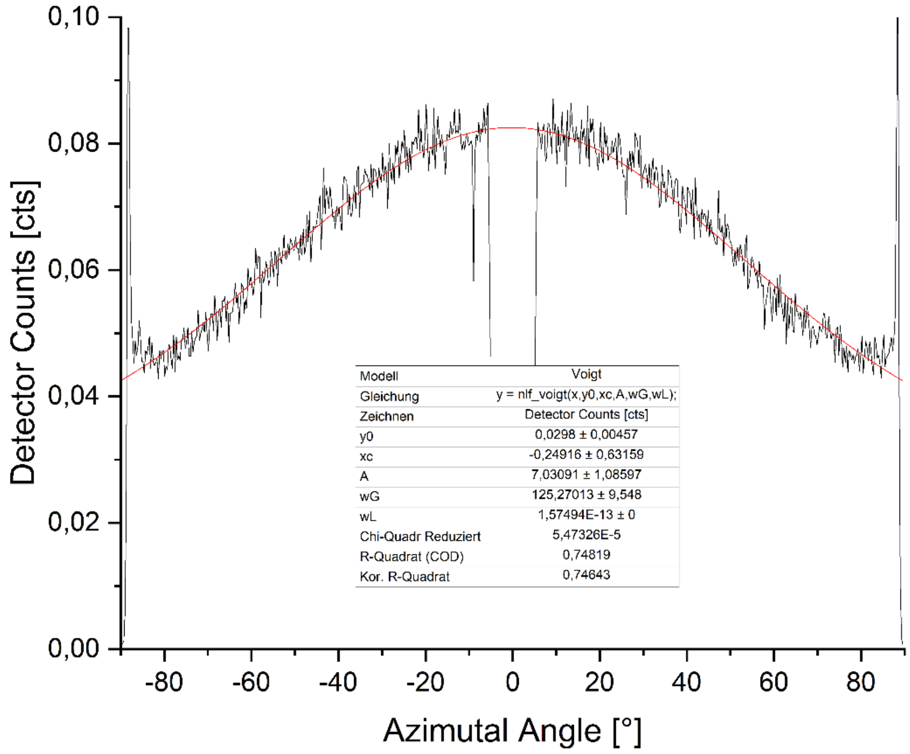

Figure S1b: CuGA@SiO_2, ϕ = 30°

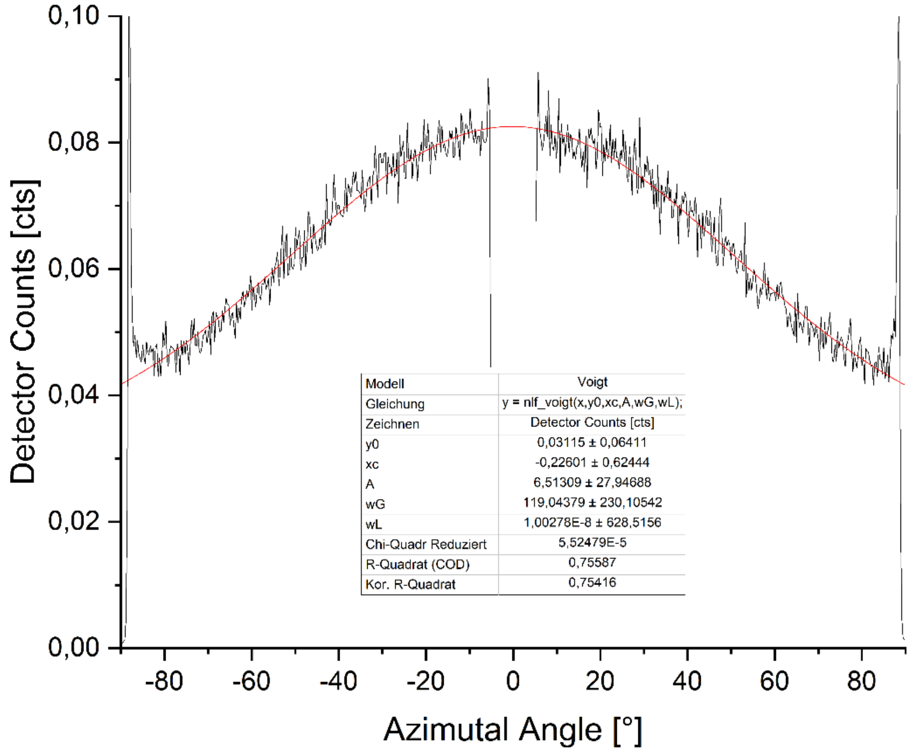

Figure S1c: CuGA@SiO_2, ϕ = 45°

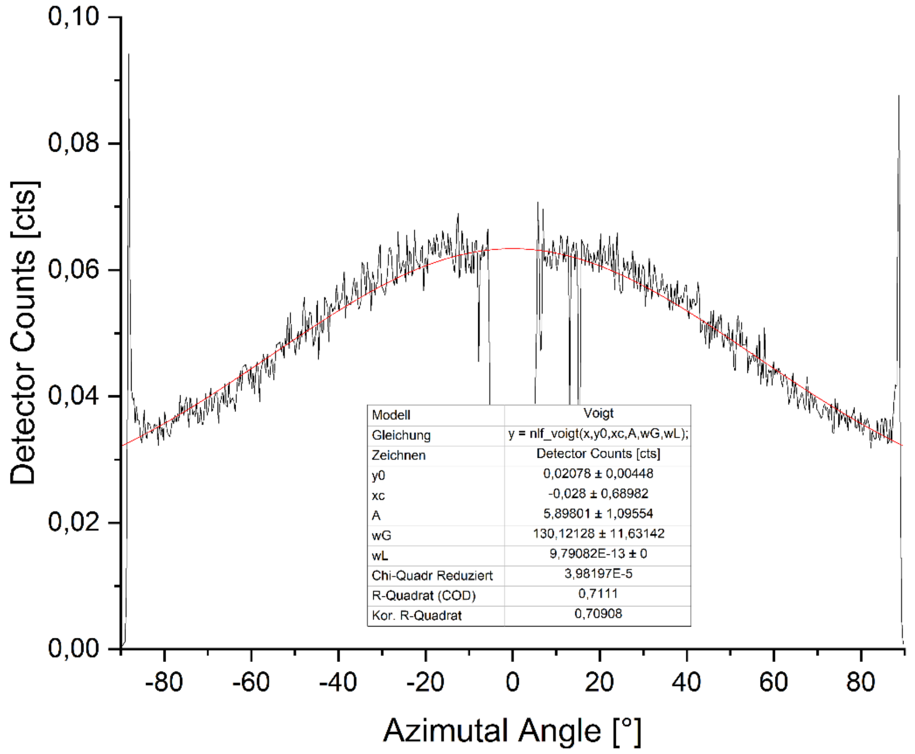

Figure S1d: CuGA@SiO_2, ϕ = 90°

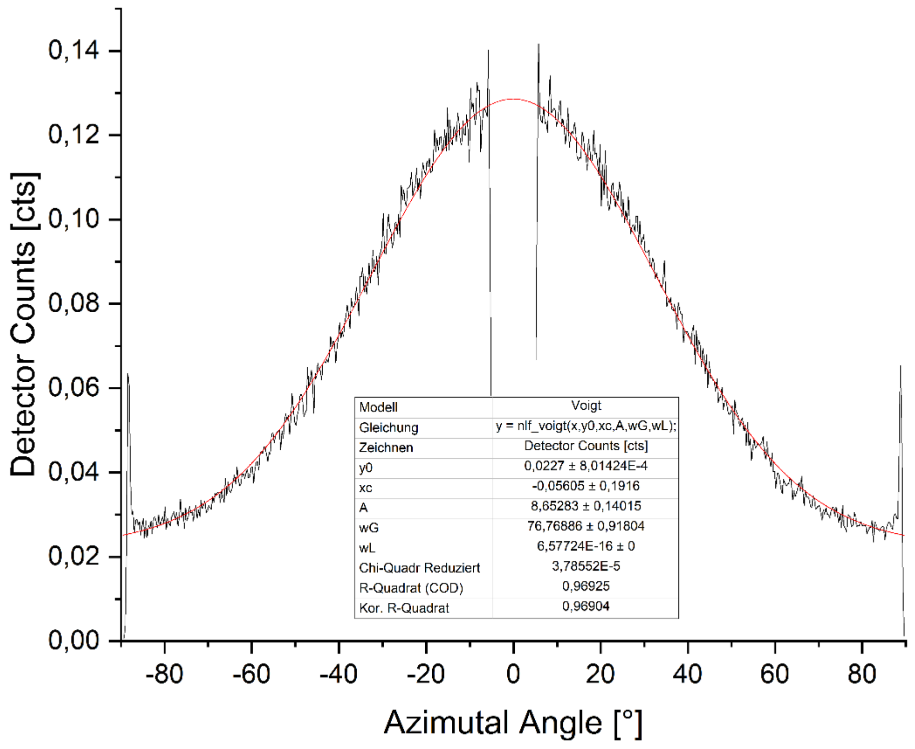

Figure S1e: CuGA@cellulose, ϕ = 0°

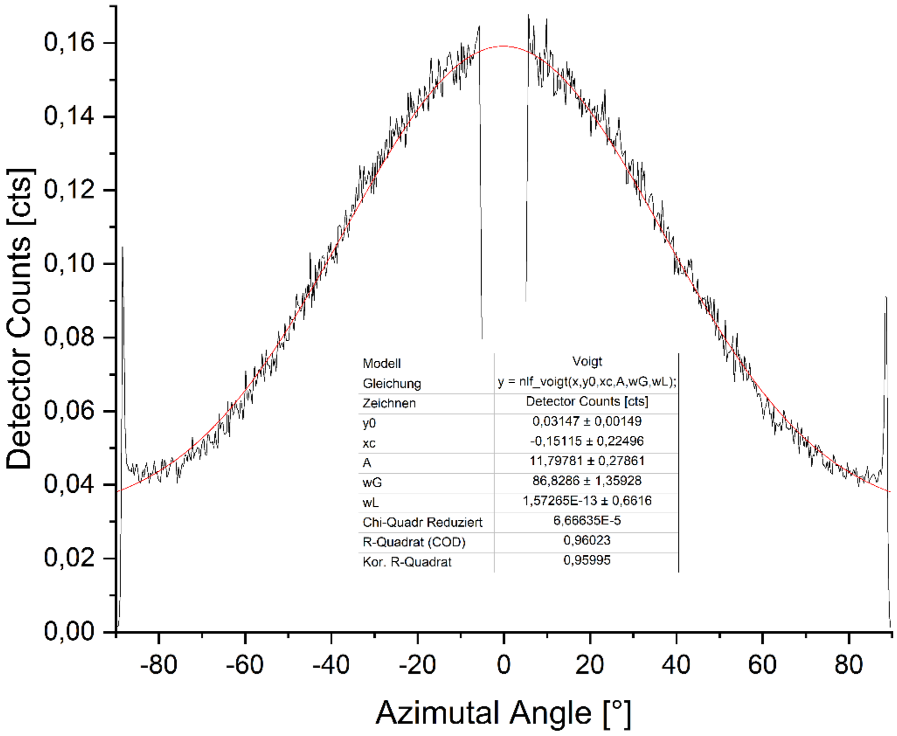

Figure S1f: CuGA@cellulose, ϕ = 45°

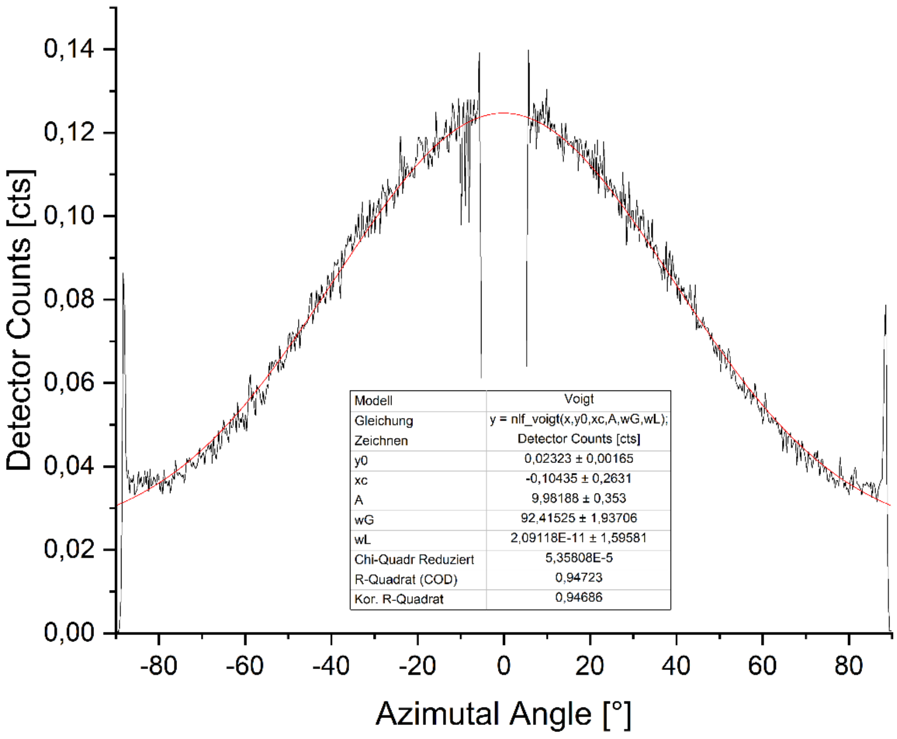

Figure S1g: CuGA@cellulose, ϕ = 90°

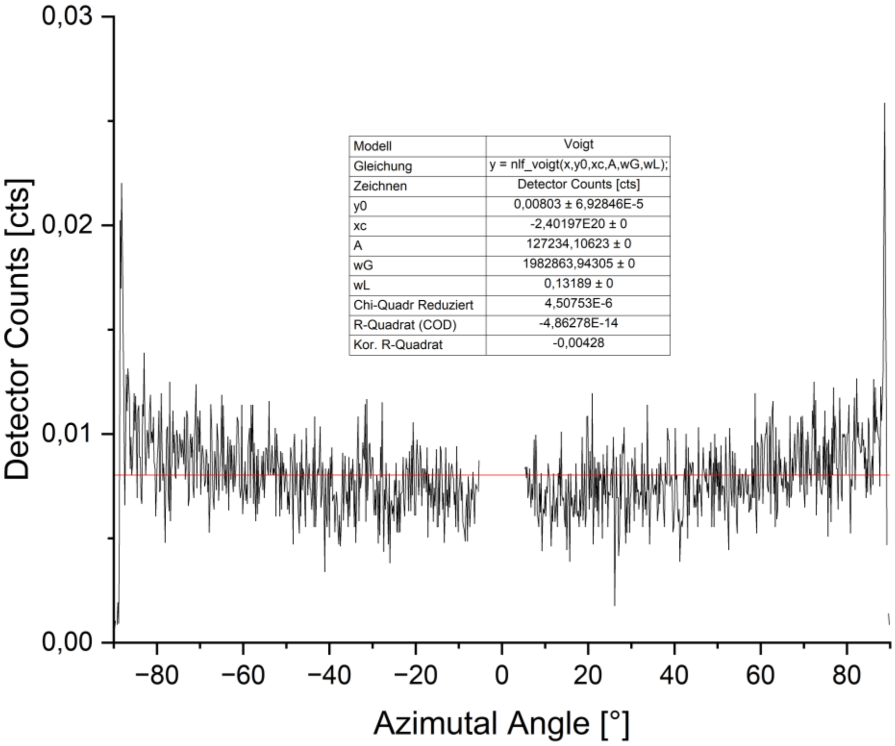

Figure S1h: CuGA@cellulose caffeate, ϕ = 0°

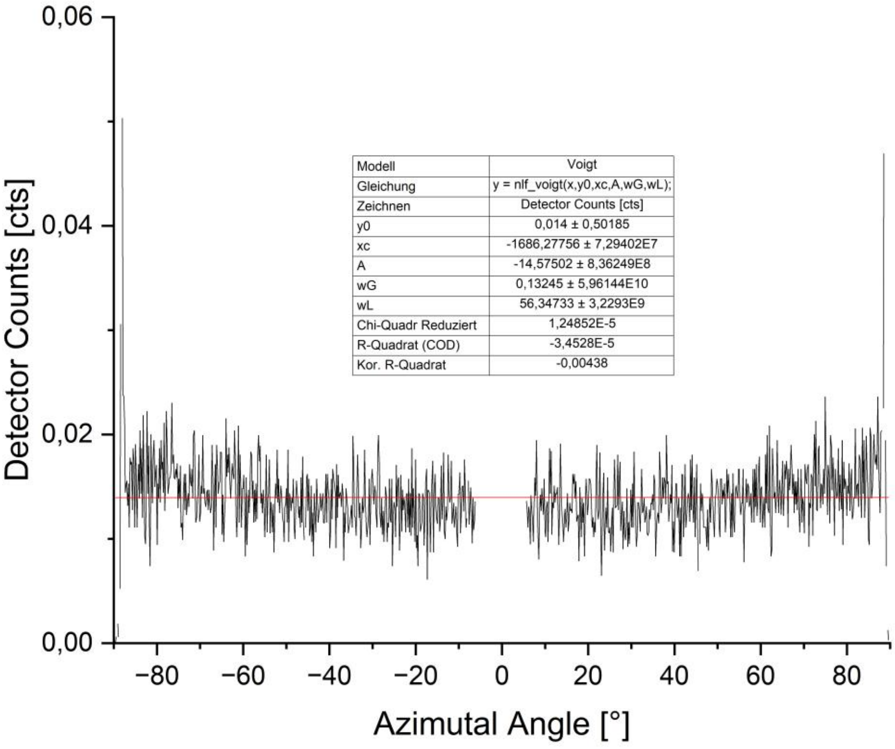

Figure S1i: CuGA@cellulose protocatechuate, ϕ = 0°

Figure S2:

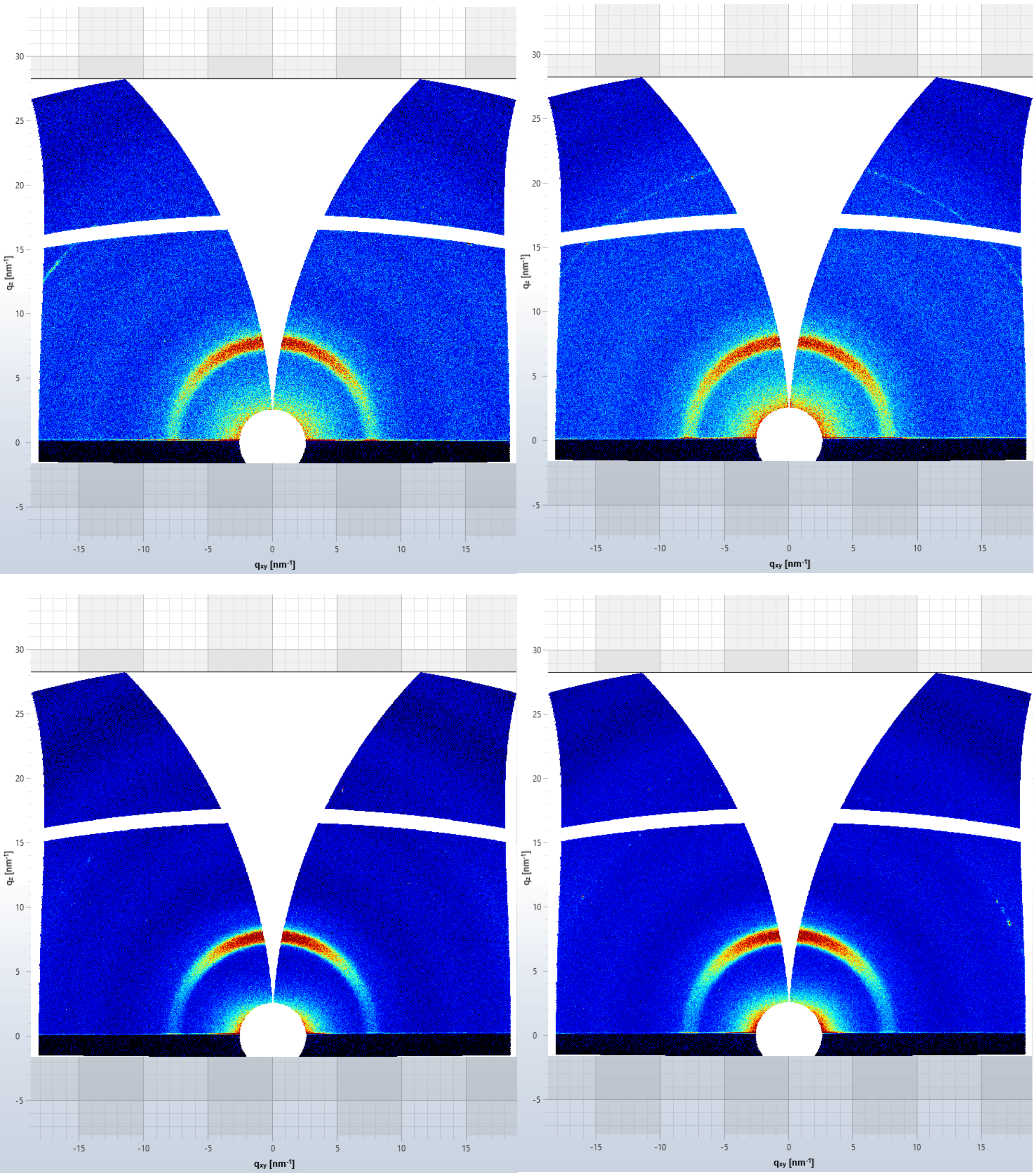

GIWAXS patterns of copper gallate (CuGA) SURMOFs on SiO_2 surfaces (top) and pure cellulose films (bottom) at $\phi = 0°$ (left) and $\phi = 45°$ (right).

Figure S3:

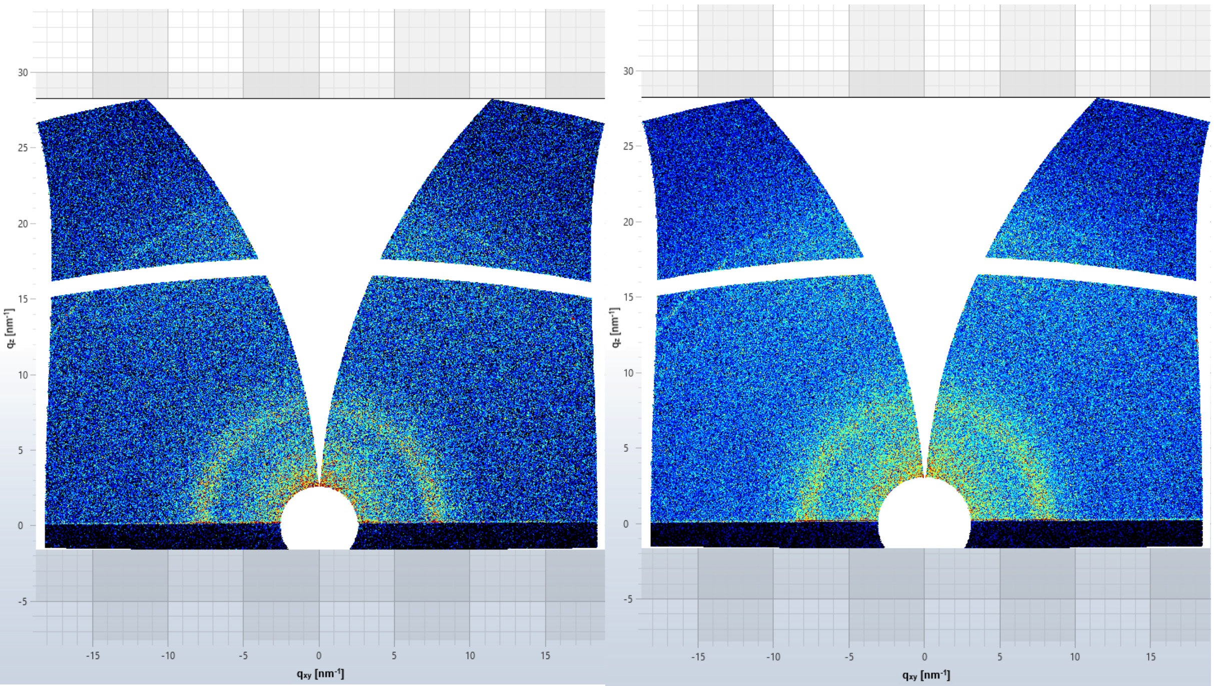

GIWAXS patterns of copper gallate (CuGA) SURMOFs on cellulose caffeate (left) and cellulose protocatechuate films (right) at ϕ = 0°.
